# Synthesis, inhibition effects and quantum chemical studies of a novel coumarin derivative on the corrosion of mild steel in a hydrochloric acid solution

**DOI:** 10.1186/s13065-016-0170-3

**Published:** 2016-04-27

**Authors:** Khalida F. Al-Azawi, Shaimaa B. Al-Baghdadi, Ayad Z. Mohamed, Ahmed A. Al-Amiery, Talib K. Abed, Salam A. Mohammed, Abdul Amir H. Kadhum, Abu Bakar Mohamad

**Affiliations:** University of Technology (UOT), Baghdad, 10001 Iraq; Department of Chemical and Process Engineering, Universiti Kebangsaan Malaysia (UKM), 43000 Bangi, Selangor Malaysia; Faculty of Engineering, University of Nizwa, 616 Nazwa, Sultanate of Oman

**Keywords:** (thiadiazol-5-yl)methoxy)coumarin, Corrosion inhibitor, Isotherm, Weight loss

## Abstract

**Background:**

The acid corrosion inhibition process of mild steel in 1 M HCl by 4-[(2-amino-1, 3, 4-thiadiazol-5-yl)methoxy]coumarin (ATC), has been investigated using weight loss technique and scanning electron microscopy (SEM). ATC was synthesized, and its chemical structure was elucidated and confirmed using spectroscopic techniques (infrared and nuclear magnetic resonance spectroscopy).

**Findings:**

The results indicated that inhibition efficiencies were enhanced with an increase in concentration of inhibitor and decreased with a rise in temperature. The adsorption equilibrium constant (K) and standard free energy of adsorption (ΔG_ads_) were calculated. Quantum chemical parameters such as highest occupied molecular orbital energy, lowest unoccupied molecular orbital energy (EHOMO and ELUMO, respectively) and dipole moment (μ) were calculated and discussed. The results showed that the corrosion inhibition efficiency increased with an increase in both the EHOMO and μ values but with a decrease in the ELUMO value.

**Conclusions:**

Our research show that the synthesized macromolecule represents an excellent inhibitor for materials in acidic solutions. The efficiency of this macromolecule had maximum inhibition efficiency up to 96 % at 0.5 mM and diminishes with a higher temperature degree, which is revealing of chemical adsorption. An inhibitor molecule were absorbed by metal surface and follow Langmuir isotherms low and establishes an efficient macromolecule inhibitor having excellent inhibitive properties due to entity of S (sulfur) atom, N (nitrogen) atom and O (oxygen) atom.

## Background

It is very important to use corrosion inhibitors to prevent metal dissolution and minimize acid consumption [1–4]. The majority of well-known acid inhibitors are organic compounds that contain nitrogen, sulfur and oxygen atoms. The inhibitory action exercised by organic compounds on the dissolution of metallic species is normally related to adsorption interactions between the inhibitors and the metal surface. The planarity (p) and lone pairs of electrons present on N, O and S atoms are important structural features that control the adsorption of these molecules onto the surface of the metal [5–7]. The effective and efficient corrosion inhibitors are those compounds that have π-bonds, contain hetero-atoms such as sulfur, nitrogen, oxygen and phosphorous and allow the adsorption of compounds on the metal surface [8–11]. The organic inhibitors decrease the corrosion rate by adsorbing on the metal surface and blocking the active sites by displacing water molecules, leading to the formation of a compact barrier film on the metal surface. Coumarins exhibit pharmacological activities, such as anticancer, anti-inflammatory [12], anti-influenza, antituberculosis [13], anti-HIV, antiviral, antialzheimer and antimicrobial activities [14]. Nowadays researchers go for coumarins to used as corrosion inhibitors due to the electronic structure, planarity, lone pairs of electrons present on oxygen and stability [15–17]. The successful control of corrosion develops the life of mechanical hardware. Nowadays corrosion inhibitors have more significant, due to their usage in industries. Organic inhibitors considered as eco-friendly much more than inorganic one. Organic inhibitors decreasing the corrosion rate by adsorbing onto the surface of the metal through the active sites namely phosphorus, sulfur, oxygen, nitrogen atoms or pi-bonds [18]. Recently the quantum chemical computations based on density function theory (DFT) become powerful investigation theoretical tool for researchers to investigate the ability of organic molecules as corrosion inhibitions. This tool offers a glance at physical insights on corrosion inhibition mechanisms [19]. In continuation of previous work [20–27], we focus herein on the design our approach to increase the inhibitive properties based on conjugated system and electron density, in addition to applied the theoretical studies to associate the inhibitive properties with electronic structures. Initially we were starting from 4-hydroxycoumarin as starting material for the synthesis of 4-[(2-amino-1, 3, 4-thiadiazol-5-yl)methoxy]coumarin (ATC) contain 1, 3, 4-thiadiazol moiety.

## Methods

### Chemistry

The chemicals utilized were supplied by Sigma-Aldrich and the purity checked by TLC (thin layer chromatography). Infrared spectra were obtained on a Thermo Scientific, NICOLET 6700 FTIR spectrometer. Nuclear magnetic resonance spectra were obtained on a JEOL JNM-ECP 400. Elemental microanalysis, was carried out using a model 5500-Carlo Erba C.H.N elemental analyzer.

### Synthesis of corrosion inhibitor “4-[(2-amino-1, 3, 4-thiadiazol-5-yl)methoxy]coumarin (ATC)”

This compound was synthesized in good yield according to the previously described procedures [28, 29]. Phosphorus oxychloride (20 ml) was added to 2-(2-oxo-2H-chromen-4-yloxy) acetic acid (0.05 mol) and the mixture was stirred for I h at room temperature. Thiosemicarbazide (4.56 g, 0.05 mol) was added and the mixture was heated and reflux for 5 h. On cooling, the mixture was poured on to ice. After 4 h stir for 15 min to decompose the excess phosphorusoxychloride, then heated under reflux for 30 min, cooling, the mixture was neutralized by 5 % potassium hydroxide, the precipitated was filtered, washed with water, dried and crystallized. Recrystallization from dichloromethane yields 55 %, m.p. 99 °C; 1H-NMR (CDCl3): δ 5.62 (s, 1H, –C=C–H), δ 4.91 and δ 5.33 (d, 2H, t, 2H, for OCH2), δ 7.23–7.87 (m, 1H, C–H aromatic ring), δ5.21 (s, NH2); IR: 3314.5 and 3375.1 cm^−1^ (s, H, amine), 291.2 (C–H alkane); 3079.1 (C–H aromatic),1752.3 cm^−1^ (C=O, lactone), 1591.1 cm^−1^(C=N, imine), 1635.3 cm^−1^ (C=C aromatic); Anal. Calcd. for C12H9N3O3S: C 52.36 %, H 3.30 %, N 15.26 %. Experimentally: C 51.64 % H 2.92 % and N 14.94 %s.

### Gravimetric information

#### Specimens

Mild steel specimens utilized throughout our work were supplied from “Metal-Samples-Company” (St. Marys, PA, United States). The weight composition percentages of the MS were: Iron, 99.21; Carbon, 0.21; Silicon, 0.38; Phosphorous, 0.09; S, 0.05; Manganese, 0.05; and Alaminuim, 0.01. The specimens were cleaned using the chemical *cleaning procedures* described in *ASTM* G1-03 test method [30]. All experiments were done in aerated and non stirred hydrochloric acid mediums contain various concentrations of (ATC).

#### Weight loss techniques

The MS specimens were suspended separately in duplicate in 200 mL of the test solution, with and without various concentrations (0.0, 0.05, 0.1, 0.15, 0.20, 0.25 and 0.50 mM) of the ATC. After 1, 2, 3, 4, 5, 10 and 24 h., of immersion time, at temperatures, namely, 303, 313, 323 and 333 K. The specimens were taken out, washed, dried, and weighed accurately. The inhibition efficiencies (% IE) values were calculated using of in Eq. 1.

1 $$ IE\left( \% \right) = \frac{{w_{o} - w_{1} }}{{w_{o} }} \times 100 $$

where, ${\text{w}}_{\text{o}}$ is the weight loss value in the absence of ATC, and ${\text{w}}_{1}$ is the weight loss values in the presence of ATC.

The corrosion rates (CR) were determined by using Eq. (2) [31, 32]

2 $$ C_{R} = \frac{87.6w}{at\rho } $$

#### Quantum chemical calculations

The molecular optimization was carried out using the density function theory (DFT)/B3LYP with basis set 6-31G. Quantum chemical calculations such as E _HOMO_ (highest occupied molecular orbital energy), E _LUMO_ (lowest unoccupied molecular orbital energy) and μ (dipole moment) were calculated and discussed.

## Results and discussion

### Weight loss method

#### Effect of *concentration*

**Fig. 1:**
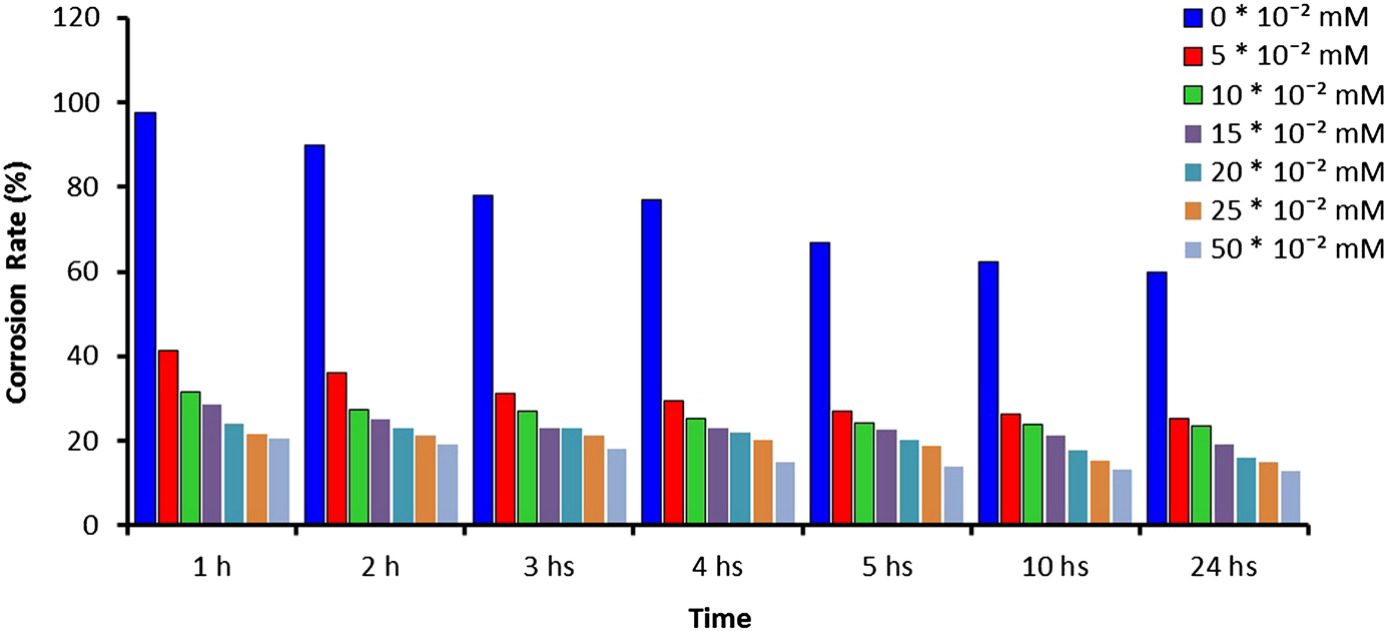
Influences of concentrations vs time for ATC on corrosion rate at 303 K

**Fig. 2:**
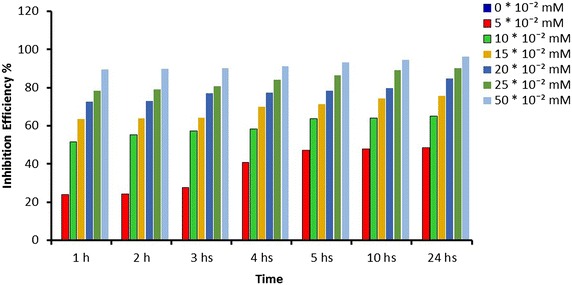
Influences of concentrations vs time for ATC on corrosion efficiencies at 303 K

Corrosion rate inhibition efficiencies were calculated for various concentrations of ATC for the duration 1, 2, 3, 4, 5, 10 and 24 h, at 303 K are shown in Figs. 1 and 2. ATC obviously diminutive the corrosion in acidic solutions for MS. The IE (%) raise with the increment of concentration of ATC and become the maximum at the highest concentration of ATC. The increment of inhibition efficiencies with the concentration imply the increase in the ATC as a potent of protection efficiency. This might be due to the adsorption of inhibitor molecule on the metal surface as a protective layer giving high inhibition efficiency. Moreover, ATC has different active sites due to N, O and S atoms that make complexation with the metal easy and that would increase its adsorption on the metal surface.

#### Effect of temperature

**Fig. 3:**
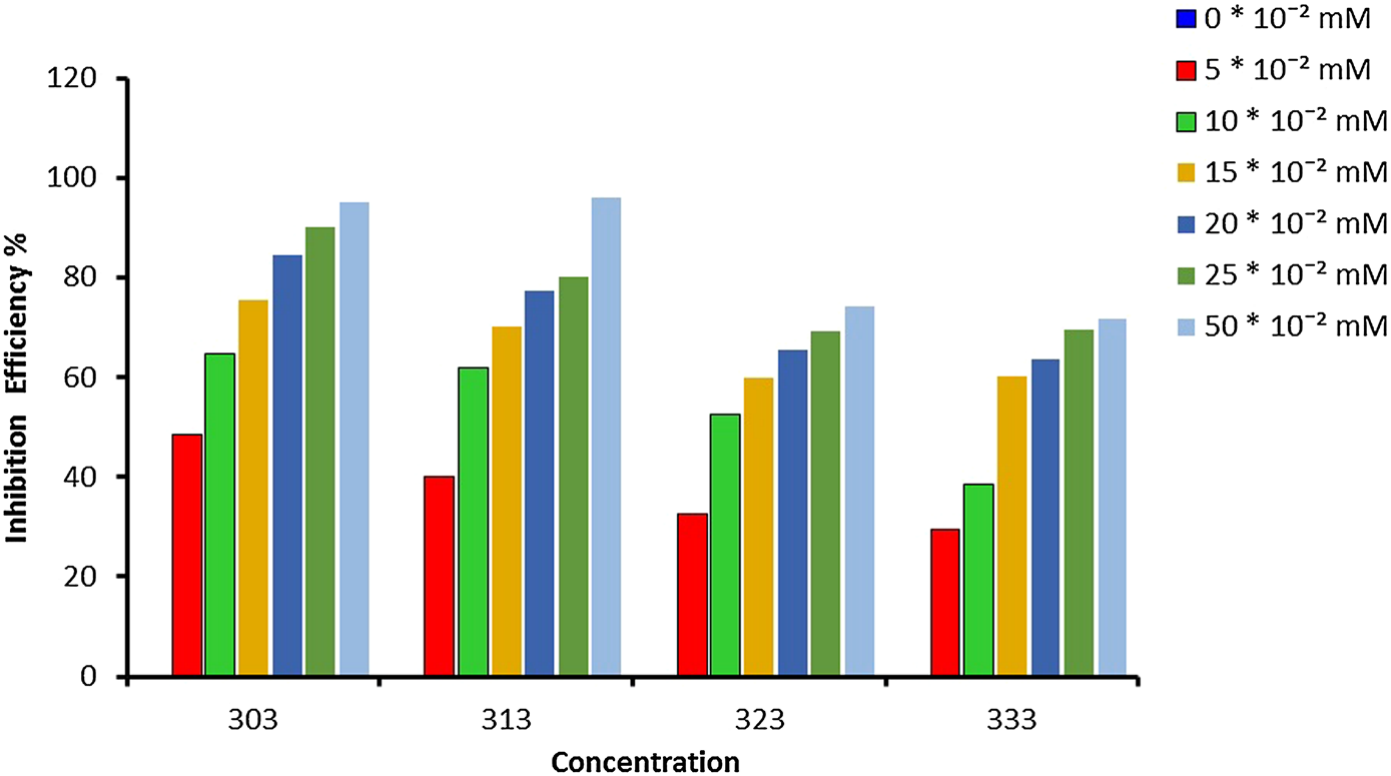
Influences of concentrations vs temperatures for ATC on corrosion efficiencies at fixed time

A differentiation of the inhibition efficiencies of ATC on mild steel in acidic medium with and without of different concentrations of ATC at various temperatures (303, 313, 323 and 333 K) indicates that corrosion efficiency rise with increasing of concentration and reduced with temperature rise (Fig. 3). Generally when the organic compounds adsorbed, the heat of adsorption will be negative, and this mean the process was an exothermic, so this is why the inhibitor efficiencies reduces when the temperature rise.

### Scanning electron microscopy, SEM

**Fig. 4:**
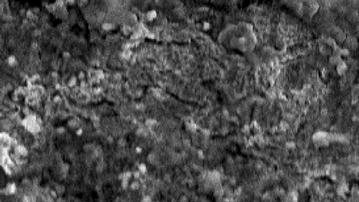
The SEM micrograph for MS in in acidic medium in absence of ATC

**Fig. 5:**
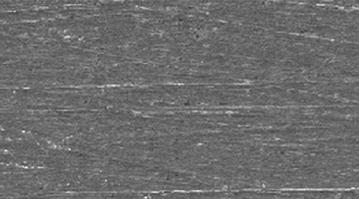
The SEM micrographs, for MS in acidic medium with 0.5 mM of the corrosion inhibitor at 30 °C for 5 h as immersion time in presence of ATC

As shown in Fig. 4, metal surface, that was originally smooth and neat, crumbled from corrosion and turn into rough surface and was extremely damaged by acidic solution. From Fig. 5, the healed surface of the metal was not suffering from remarkable corrosion. The synthesized macromolecule that supply protection to the surface of the metal from the acid.

### Adsorption isotherm and mechanism of corrosion and inhibition

Generally, IE of corrosion inhibitors depend on the adsorption coefficient of MS. The stabilization of the adsorbed inhibitor molecules differ according to the type of adsorption is chemical or physical or both. Turn out it is needful to explore the interaction between the metal and the inhibitor through adsorption isotherms [33]. The action of corrosion inhibitor over MS surface can be interpreted according to adsorption isotherm. Generally adsorption be based on the morphology nature. The mechanism of adsorption of organic molecules on MS surface can be clarified by means of the investigation of adsorption isotherm and adsorptive conduct of the inhibitor. Langmuir, Frumkin, Temkin and Freundluich isotherms were the most considerably utilized adsorption isotherm [34]. The corrosion inhibitors of natural and synthetic organic inhibitors on MS in acidic medium can be showed by a molecular adsorption technique. The process of adsorption was impacted by the structures and nature of the molecules in addition to the nature of the surface/charged metals and the types of media used [35, 36]. Surface coverage (θ) for the various concentrations of the tested inhibitor was utilized to elucidate the preferable adsorption isotherm to determine the adsorption process. To estimated θ, it was proposed [37] that the inhibition efficiency is due fundamentally to the blocking effect of the adsorbed molecules or ions and so, Eq. (3) will be applied.

3 $$ {{\uptheta }} = \frac{{{\text{IE}}\% }}{100} $$

The plot of $\frac{{{\text{C}}_{\text{inh}} }}{\theta }$ vs concentration of inhibitor ($C_{inh}$) produce a straight line with an approximately unit slope, indicating that the inhibitor under study obeys the Langmuir adsorption isotherm [38], as in the Eq. (4).

4 $$ \frac{{C_{\text{inh}} }}{\theta } = \frac{1}{{K_{ads} }} + C_{inh } $$

$K_{ads}$ is the adsorption constant obtained from the intercept of the straight line.

Equation 5 give the association of the intercept of the straight line $K_{ads}$ with the standard free energy $\Updelta G_{ads}^{^\circ }$

5 $$ \Updelta G_{ads}^{^\circ } = - RTln\left[ {55.5K_{ads} } \right] $$

whereas R is the universal gas constant, the number 55.5 is the molar concentration of water in solution and T is the absolute temperature.

**Fig. 6:**
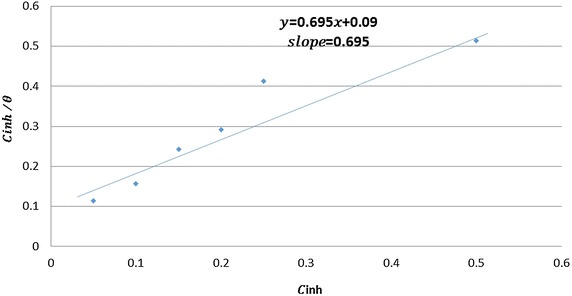
Linear equation

From Fig. 6 we can calculate $K_{ads}$ and $\Updelta G_{ads}^{^\circ }$. $\Updelta G_{ads}^{^\circ }$.

From Fig. 6$\Updelta G_{ads}^{^\circ }$ was calculated and it was −31.51 kJ/mol. The negatively charge for $\Updelta G_{ads}^{^\circ }$elucidate the natural adsorption of the ATC on the MS surface and the vigorous interaction through the ATC and MS surface. Generally, if $\Updelta G_{ads}^{^\circ }$ is nearly −20 kJ/mol then it appropriate with physical adsorption, while if $\Updelta G_{ads}^{^\circ }$ nearly −40 kJ/mol then it is chemical adsorption occurring with the sharing of electrons from molecules of the inhibitor to the MS surface. In our work the $\Updelta G_{ads}^{^\circ }$ is around −40 kJ/mol and demonstrate mechanism of adsorption of ATC by means of chemical adsorption [39]. In hydrochloric acid solution the following mechanism is proposed for the corrosion of mild steel [40]. The anodic dissolution mechanism of mild steel is

$$ {\text{Fe}} + {\text{Cl}}^{ - } \leftrightarrow ({\text{FeCl}}^{ - } )_{\text{ads}} $$

$$ ({\text{FeCl}}^{ - } )_{\text{ads}} \leftrightarrow ({\text{FeCl}}^{ + } )_{\text{ads}} + {\text{e}}^{ - } $$

$$ ({\text{FeCl}}^{ + } )_{\text{ads}} \leftrightarrow {\text{Fe}}^{ + + } + {\text{Cl}}^{ - } $$

The cathodic hydrogen evolution mechanism is

$$ {\text{Fe}} + {\text{H}}^{ + } \leftrightarrow ({\text{FeH}}^{ + } )_{\text{ads}} $$

$$ ({\text{FeH}}^{ + } )_{{{\text{ads}}}}  + {\text{e}}^{ - } ({\text{FeH}})_{{{\text{ads}}}} $$

$$  ({\text{FeH}}^{ + } )_{{{\text{ads}}}}  + {\text{H}}^{ + }  + {\text{e}}^{ - }  \to {\text{Fe}} + {\text{H}}_{2}  $$

Generally, the corrosion inhibition mechanism in an acid medium is adsorption of the inhibitor on the metal surface. The process of adsorption is influenced by different factors like the nature and charge of the metal, the chemical structure of the organic inhibitor and the type of aggressive electrolyte [41–43].

### Suggested mechanisms of actions of coumarin as inhibitor

**Fig. 7:**
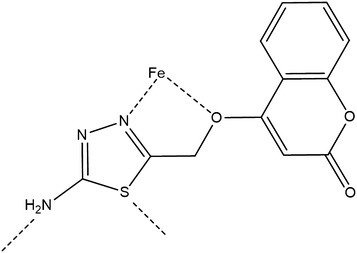
The suggested mechanism of action of the ATC as corrosion inhibitor

Chemically the inhibitor is adsorbed on the metal surface and forms a protective thin film or chemical bonds form by reaction between the inhibitor and metal. The adsorption mechanism of organic inhibitors can proceed via one of these routes. 1st, charged molecules and metal attract electrostatically. 2nd, the interaction between unpaired electrons and the metal surface. 3rd, interaction between π-electrons and the metal surface. Organic inhibitors protect the metal surface by blocking cathodic or anodic reactions or both and forming insoluble complexes. The inhibition efficiency of our corrosion inhibitor against the corrosion of mild steel in 1 M hydrochloric acid can be explained according to the number of adsorption sites, charge density, molecular size, mode of interaction with the metal surface and ability of formation of metallic insoluble complex. The π electrons for the double bonds and free electrons on the oxygen and nitrogen atoms form chemical bonds with the metal surface as shown in Fig. 7.

### Quantum chemical calculations

**Fig. 8:**
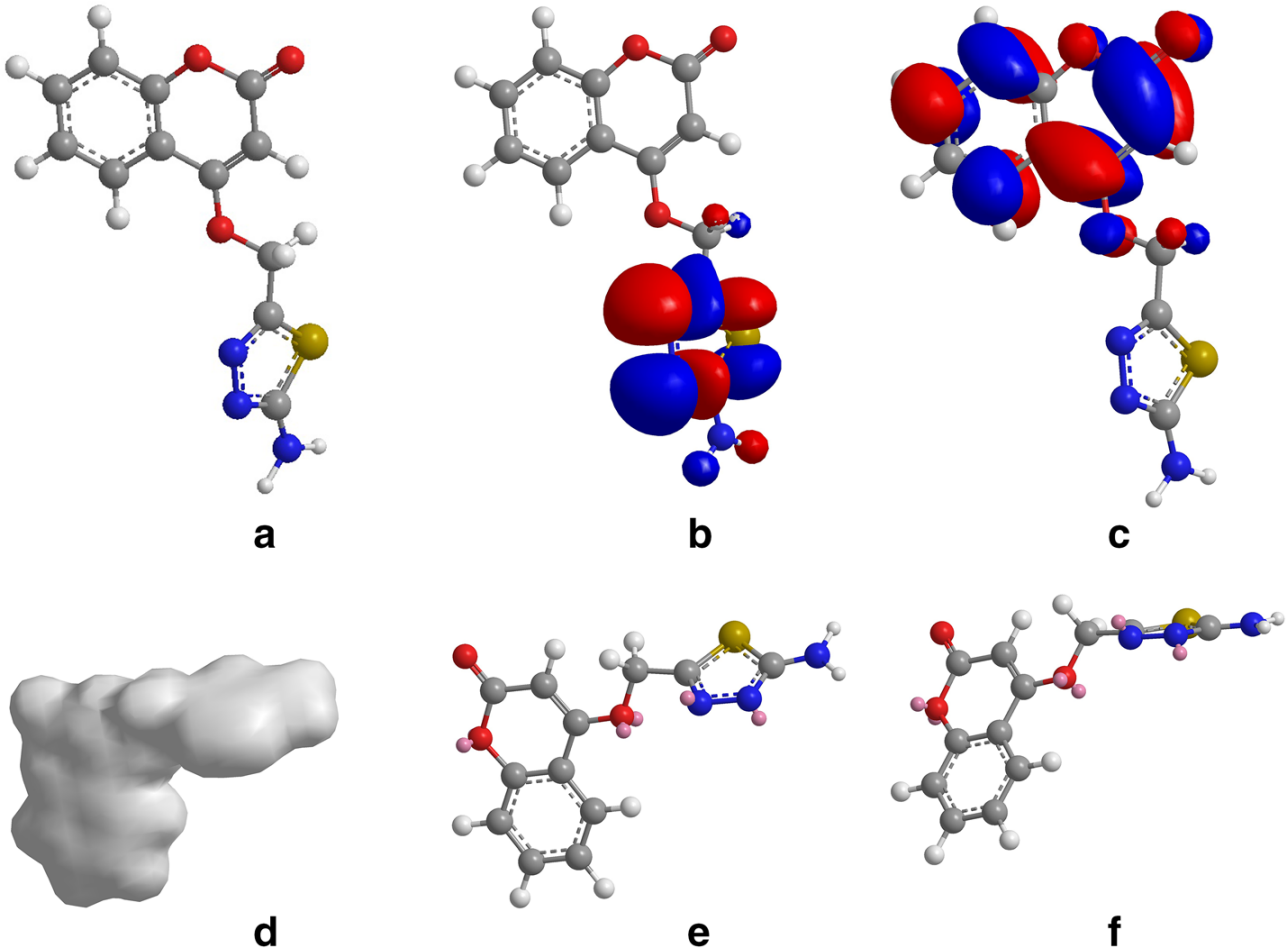
Electronic properties of (**a**) 3d-structure of ATC; (**b**) HOMO orbital; (**c**) LUMO orbital; (**d**) total electron density; (**e**) Fukui (f−) function; **f** Fukui (f+) function

The structural nature of the organic corrosion inhibitor and inhibition mechanism can be described by density functional theory (DFT). This technique has been found to be successful in providing insights into the chemical reactivity and selectivity in terms of global parameters such as electro-negativity (v), hardness (g) and softness (S), and local softness (sđ~r Þ) [44, 45]. The design of the (ATC), for use as a corrosion inhibitor was based on several factors. First, the molecule contains oxygen, nitrogen and sulfur atoms as active centers. Second, (ATC), can be easily synthesized and characterized. Third, planarity and finaly the resonance structure of (ATC). Excellent corrosion inhibitors are usually organic compounds that not only offer electrons to unoccupied orbitals of the metal but also accept free electrons from the metal [46]. Quantum chemical theoretically calculations were used to investigated the interactions between metal and inhibitor [47]. Highest occupied molecular orbital (HOMO), lowest unoccupied molecular orbital (LUMO), and Fukui functions as well as the total electron density of (ATC), are presented in Fig. 7. The blue and red iso-surfaces depict the electron density difference; the blue regions show electron accumulation while the red regions show electron loss. Quantum parameters such as EHOMO, EHOMO and dipole moment are provided in Table 1. The HOMO regions for the molecule, which are the sites at which electrophiles attack and represent the active centers with the utmost ability to interact with the metal surface atoms, has contributions from carbonyl, methanimine and amine. On the other hand, the LUMO orbital can accept electrons from the metal using anti-bonding orbitals to form feedback bonds are saturated around the coumarin ring [48]. Correspondingly, a high value of the HOMO energy (EHOMO) indicates the tendency of a molecule to donate electrons to an appropriate acceptor molecule with low energy or an empty electron orbital, whereas the energy of the LUMO characterizes the susceptibility of molecule toward nucleophilic attack [49]. Low values of the energy of the gap ΔE = ELUMO−EHOMO implies that the energy to remove an electron from the last occupied orbital will be minimized, corresponding to improved inhibition efficiencies [50]. EHOMO value (Table 1) do not vary very significantly for (ATC), which means that any observed differences in the adsorption strengths would result from molecular size parameters rather than electronic structure parameters. The seemingly high value of ΔE is in accordance with the nonspecific nature of the interactions of the molecule with the metal surface. A relationship between the corrosion inhibition efficiency of the (ATC), with the orbital energies of the HOMO (EHOMO) and LUMO (ELUMO) as well as the dipole moment (μ) is shown in Table 1. As is clearly observed, the inhibition efficiency increases with an increase in EHOMO values along with a decrease in ELUMO values. The increasing values of EHOMO indicate a higher tendency for the donation of electrons to the molecule with an unoccupied orbital. Increasing values of EHOMO thus facilitate the adsorption of the inhibitor. Thus, enhancing the transport process through the adsorbed layer would improve the inhibition effectiveness of the inhibitor. This finding can be explained as follows. ELUMO indicates the ability of the molecule to accept electrons; therefore, a lower value of ELUMO more clearly indicates that the molecule would accept electrons [51]. The direction of a corrosion inhibition process can be predicted according to the dipole moment (μ). Dipole moment is the measure of polarity in a bond and is related to the distribution of electrons in a molecule. In spite of the fact that literature is conflicting on the utilization of μ as an indicator of the direction of a corrosion inhibition reaction, it is for the most part concurred that the adsorption of polar compounds having high dipole moments on the metal surface ought to prompt better inhibition efficiency. The data obtained from the present study indicate that the (ATC), inhibitor has the value of μ = 4.959 and highest inhibition efficiency (96.0 %). The dipole moment is another indicator of the electronic distribution within a molecule. A few researchers express that the inhibition efficiency increments with increasing values of the dipole moment, which relies on upon the sort and nature of molecules considered. However, there is a lack of agreement in the literature on the correlation between μ and IE  %, as in some cases no significant relationship between these values has been identified [52, 53]. The electron density (charge distribution) is saturated all around molecule; hence we should expect flat-lying adsorption orientations [48]. The local reactivity of molecule was analyzed by means of the Fukui indices (FI) to assess reactive regions in terms of nucleophilic (f+) and electrophilic (f−) behavior. Figure 8d shows that the f− functions of molecule correspond with the HOMO locations, indicating the sites through which the molecule could be adsorbed on the metal surface, whereas f+ (Fig. 8e) correspond with the LUMO locations, showing sites through which the molecule could interact with the nonbonding electrons in the metal. High f− values are associated with the nitrogen atoms of thiadiazole ring and oxygen of the pyrone ring, in addition to oxygen atom of the bridge.

**Table 1:** Calculated quantum chemical properties for the most stable conformation of (ATC)

Function	Function values
EHOMO	7.909 eV
ELUMO	3.901 eV
EHOMO–ELUMO	−4.008 eV
*f* ^*−*^ *max*	0.164
*f* ^*+*^ *max*	0.093
Dipole Moment	4.959

### Mulliken charge

**Fig. 9:**
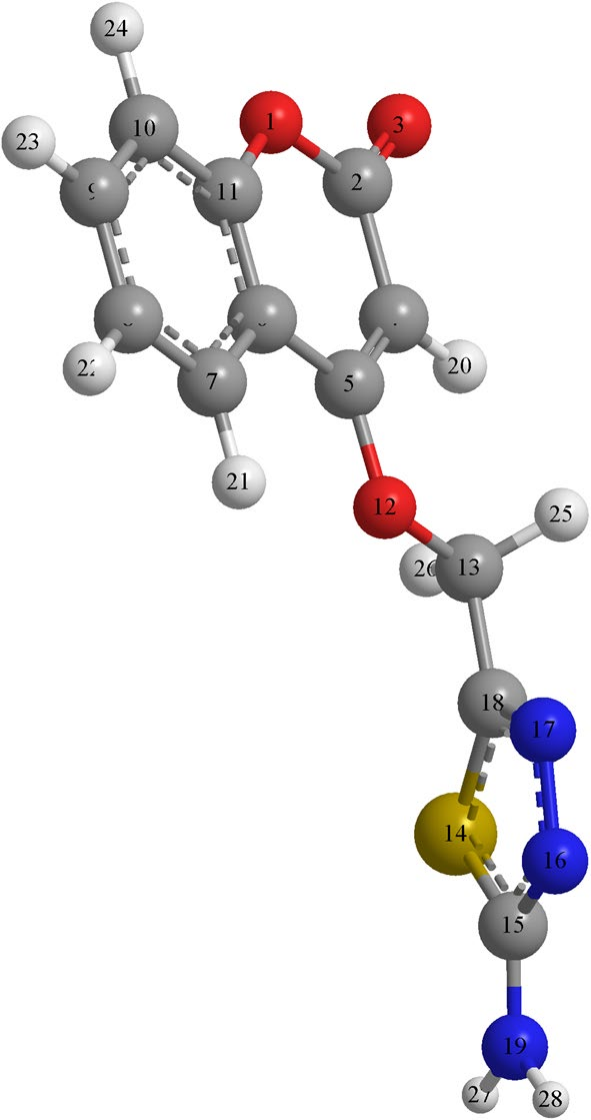
The three dimensional structure of ATC

The Mulliken charge distribution of (ATC) is presented in Table 2. Atom can be easily donating its electron to the empty orbital of the metal if the Mulliken charges of the adsorbed center become more negative [54]. It could be readily observed that nitrogen, oxygen, sulfur and some carbon atoms have high charge densities. The regions of highest electron density are generally the sites to which electrophiles can attach [54, 55]. Therefore, N, O, S and some C atoms are the active centers, which have the strongest ability to bond to the metal surface. Conversely, some carbon atoms carry positive charges, which are often sites where nucleophiles can attach. Therefore, (ATC) can also accept electrons from Fe through these atoms. It has been reported that excellent corrosion inhibitors can not only offer electrons to unoccupied orbitals of the metal but also accept free electrons from the metal [56]. According to the description of frontier orbital theory, HOMO (Fig. 9) is often associated with the electron donating ability of an inhibitor molecule. The molecules have tendency to donate electrons to a metal with empty molecule orbital if they have high EHOMO values. ELUMO, conversely, indicates the ability of the molecule to accept electrons [57]. Acceptance of electrons from a metal surface is easier when the molecule has lower value of ELUMO. The gap between the LUMO and HOMO energy levels of inhibitor molecules is another important parameter. Low absolute values of the energy band gap (E = ELUMO−EHOMO) mean good inhibition efficiency [58].

**Table 2:** Charges (Mulliken charges) for the ATC

Atom	Charge	Atom	Charge	Atom	Charge	Atom	Charge
O(1)	−0.2442	C(8)	−0.1049	C(15)	−0.0994	H(22)	0.0689
C(2)	0.3942	C(9)	−0.0009	N(16)	−0.1540	H(23)	0.0680
O(3)	−0.3089	C(10)	−0.0871	N(17)	−0.0458	H(24)	0.0876
C(4)	−0.2699	C(11)	0.1634	C(18)	−0.2814	H(25)	0.0382
C(5)	0.2471	O(12)	−0.2770	N(19)	−0.2055	H(26)	0.0115
C(6)	−0.1560	C(13)	0.2660	H(20)	0.0977	H(27)	0.1429
C(7)	0.0340	S(14)	0.3809	H(21)	0.0738	H(28)	0.1606

## Conclusions

Our research demonstrate that the synthesized macromolecule represents an excellent inhibitor for materials in acidic solutions. The efficiency of this macromolecule had maximum inhibition efficiency up to 96 % at 0.5 mM and diminish with a higher temperature degree, which is revealing of chemical adsorption. Inhibitor molecules were absorbed by metal surface and follow Langmuir isotherms low and establishes an efficient macromolecule inhibitor hading excellent inhibitive properties due to entity of S (sulfur) atom, N (nitrogen) atom and O (oxygen) atom. SEM (Scanning electron microscope) measurements were confirming the figuration of a protective metal surface. Inhibition study of synthesized macromolecules obviously expose their function in the protection of MS in 1 M HCl.
